# Expression of Neurotrophin Genes in the Hypothalamus of Stressed and Allopregnanolone-Infused Sheep

**DOI:** 10.3390/ijms262010062

**Published:** 2025-10-16

**Authors:** Patrycja Młotkowska, Bartosz Osuch, Elżbieta Marciniak, Katarzyna Roszkowicz-Ostrowska, Tomasz Misztal

**Affiliations:** Department of Animal Physiology, The Kielanowski Institute of Animal Physiology and Nutrition, Polish Academy of Sciences, Instytucka 3 St., 05-110 Jablonna, Poland; p.mlotkowska@ifzz.pl (P.M.); b.osuch@ifzz.pl (B.O.); e.marciniak@ifzz.pl (E.M.); k.roszkowicz@ifzz.pl (K.R.-O.)

**Keywords:** neurotrophins, allopregnanolone, hypothalamus, stress, adult neurogenesis

## Abstract

The hypothalamus is a key regulator of fundamental physiological processes and a site of adult neurogenesis. Allopregnanolone (ALLO) is a neurosteroid that mitigates the adverse effects of stress on the central nervous system and also affects neurogenesis. This study examined the effects of acute stress and ALLO administration (separately or in combination) into the third brain ventricle on the expression of neurotrophins and Trkβ receptor in distinct hypothalamic areas of sexually active female sheep. Expression of genes encoding brain-derived neurotrophic factor (BDNF), nerve growth factor (NGF), neurotrophin-3 (NT-3), neurotrophin-4 (NT-4) and the Trkβ receptor was analyzed in the medial basal hypothalamus (MBH), arcuate nucleus (ARC), anterior hypothalamus (AHA), paraventricular nucleus (PVN), and preoptic area (POA). Acute stress stimulated the expression of neurotrophins (BDNF, NGF, and NT-3) in the ARC and PVN, while inhibitory effects predominated in the MBH, AHA and POA. ALLO alone mainly suppressed neurotrophins expression, while stimulatory effects were limited to the BDNF–Trkβ system in the ARC and Trkβ in the AHA. When combined with stress, ALLO either counteracted stress-induced increases in neurotrophins expression or produced no effect. The results demonstrate that acute stress can differentially modify neurotrophins mRNA expression in hypothalamic regions, activating neurotrophic activity in specific nuclei. The predominant inhibitory effect of ALLO on neurotrophin synthesis, particularly under conditions of acute stress, may help prevent excessive neuronal activation. Conversely, the upregulation of the BDNF-Trkβ system in the ARC indicates a positive relationship between this neurosteroid and hypothalamic adult neurogenesis.

## 1. Introduction

Neurotrophins, including brain-derived neurotrophic factor (BDNF), nerve growth factor (NGF), neurotrophin-3 (NT-3), and neurotrophin-4 (NT-4) are a family of low-molecular-weight proteins that bind to neuronal surface receptors, and activate various intracellular signaling [1,2,3]. In the adult brain, their primary role is associated with neurogenesis, a process mainly occurring in the hippocampus. Neurogenesis involves the proliferation and differentiation of new neurons from neural stem or progenitor cells, followed by their integration into neural circuits, thereby supporting neuroplasticity. Neurotrophins also facilitate brain adaptation to changing conditions and regeneration in response to injury [4]. Recent studies have indicated that adult neurogenesis occurs in the hypothalamus, with neurogenic sites mainly located in the subependymal zone of the third ventricle, particularly within the arcuate nucleus (ARC) and median eminence (ME) [5,6,7,8].

Neuroplasticity and regenerative processes in the hypothalamus are supported by BDNF—the most extensively studied neurotrophin [9]. BDNF mediates its effects primarily through the activation of tropomyosin receptor kinase β (Trkβ) [2,9,10], and is directly involved in the regulation of memory, emotions, and synaptic plasticity [11]. BDNF deficiency can lead to persistent dysfunction of brain regions controlling emotions and is a major factor in the pathophysiology of depression and anxiety disorders [12,13]. NGF also affects limbic system function, while NT-3 and NT-4 play complementary roles, supporting BDNF-Trkβ signaling [14]. Antidepressant drugs stimulate the release of BDNF and NGF and promote their signaling through Trkβ [15,16,17]. Postmortem studies also reported decreased BDNF mRNA and protein levels, along with reduced Trkβ expression in the brain of patients with depression [18,19,20] and suicide victims [21,22,23].

Neurotrophins modulate structural and functional changes in the hypothalamus in response to stress [24]. A central component of this response is the hypothalamic–pituitary–adrenal (HPA) axis. Stress-induced activation of the HPA axis triggers neurons in the paraventricular nucleus (PVN) to release corticotropin-releasing hormone (CRH), which stimulates pituitary secretion of adrenocorticotropic hormone (ACTH), and subsequent cortisol release from adrenal glands [9]. Short-term stress (e.g., 2-h immobilization) has been shown to increase BDNF mRNA levels in the PVN [25], whereas chronic stress differentially affects hypothalamic BDNF depending on its duration and type [26]. Moreover, glucocorticoids inhibit BDNF expression and its signaling function through the Trkβ receptor [25]. Studies on NGF in the PVN in adult rats found no significant changes in NGF-immunoreactive neurons after exposure to acute or chronic stress, although older rats displayed an age-related decline [27]. Defining how stress alters hypothalamic neurotrophins levels in the hypothalamus may provide important information regarding the pathophysiological mechanisms underlying stress-related mental and somatic disorders.

Both regulation of the HPA axis and modulation of neuroplasticity may be influenced by allopregnanolone (ALLO), a neuroactive metabolite of progesterone [28]. This brain-derived neurosteroid potentiates inhibitory γ-aminobutyric acid (GABA) signaling by binding to GABA_A_ receptors [29]. Through this mechanism, ALLO exerts anxiolytic, antidepressant, and neuroprotective effects [30], while also limiting excessive HPA axis activation and promoting neurogenesis [31,32]. In depressive states, especially those related to hormonal disturbances, such as postpartum depression, ALLO levels are markedly decreased. This reduction may contribute to GABA/glutamate imbalance, heightened HPA axis activity, and impaired neuroplasticity [33]. Disruption of inhibitory–excitatory balance in the CNS can also adversely affect neurotrophin levels, which support and regulate neurogenic processes [34].

Considering the strong links between adult neurogenesis and the neuroendocrine response to stress and ALLO, we tested a hypothesis that acute stressors and ALLO, both independently and in combination, could regulate the neurotrophins and Trkβ receptor transcript levels in the hypothalamus of female sheep. Analyses focused on key hypothalamic regions: the medial-basal hypothalamus (MBH), with isolated specific ARC, which regulate energy homeostasis, neuroendocrine signaling, and metabolism; the anterior hypothalamic area (AHA), involved in thermoregulation and behavioral control such as aggression and defensive responses to threats; the PVN, an integrative center coordinating neuroendocrine, autonomic, and behavioral responses to internal and external challenges, including stressors; and the preoptic area (POA), with major roles in thermoregulation, reproductive behavior, and sleep [35]. These different hypothalamic regions are expected to exhibit varying levels of neurotrophic activity in response to experimental factors. The sheep model was selected due to its slower neuronal development and longer lifespan, providing a more translationally relevant model for studying adult neurogenesis compared to rodent systems [36].

## 2. Results

**Figure 1:**
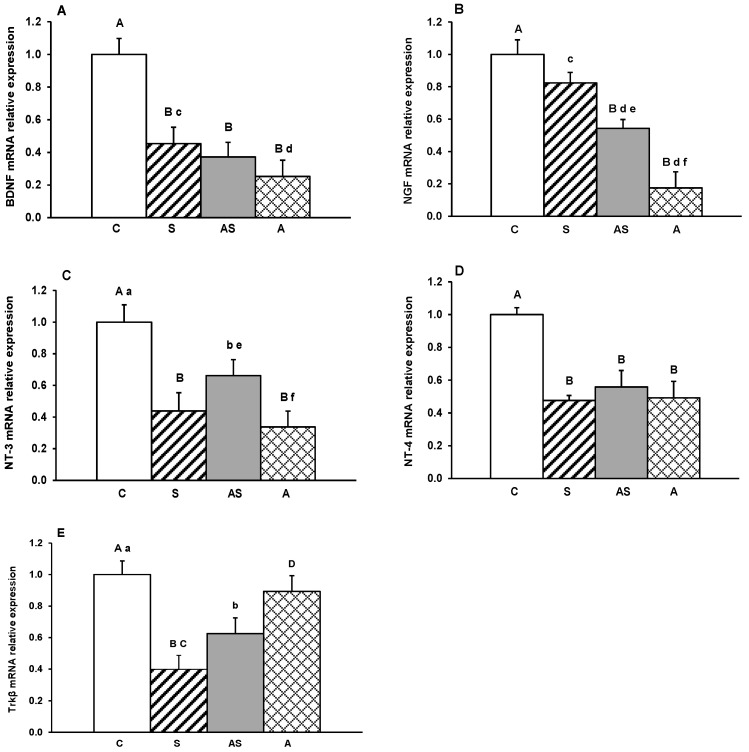
Relative mRNA expression (mean ± SEM) of brain-derived neurotrophic factor (BDNF) (**A**), nerve growth factor (NGF) (**B**), neurotrophin-3 (NT-3) (**C**), neurotrophin-4 (NT-4) (**D**) and tyrosine receptor kinase β (Trkβ) (**E**) in the medial-basal hypothalamus (MBH) of sheep treated with vehicle (C), vehicle and stressful stimuli (S), allopregnanolone and stressful stimuli (AS) and allopregnanolone alone (A). Significance of differences: ab, *p* < 0.05 and AB, *p* < 0.01 vs. control group; cd, *p* < 0.05 and CD, *p* < 0.01 vs. stressed group and ef, *p* < 0.05 vs. allopregnanolone-stressed group.

**Figure 2:**
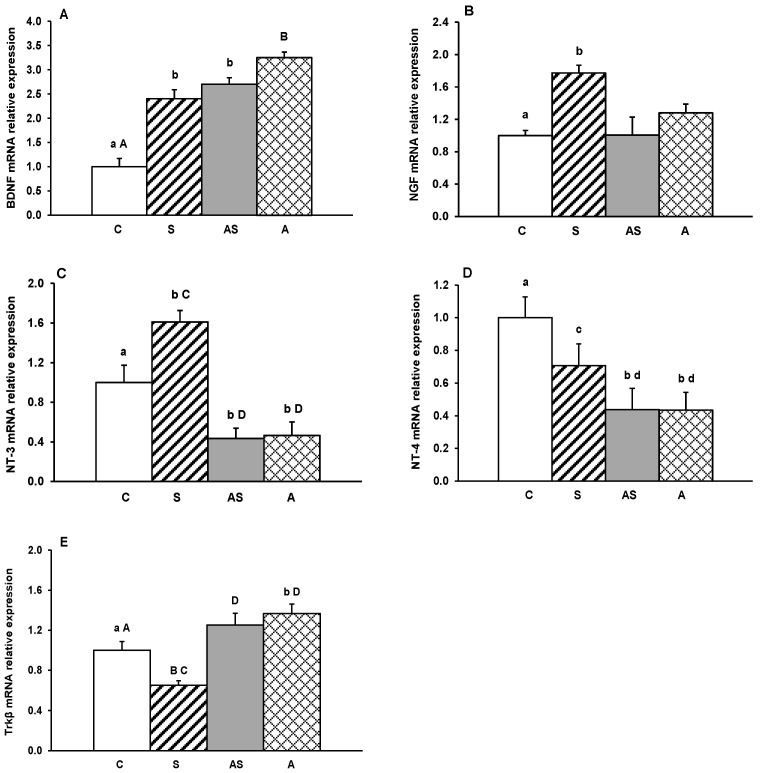
Relative mRNA abundance (mean ± SEM) of brain-derived neurotrophic factor (BDNF) (**A**), nerve growth factor (NGF) (**B**), neurotrophin-3 (NT-3) (**C**) and neurotrophin-4 (NT-4) (**D**) and tyrosine receptor kinase β (Trkβ) (**E**) in the arcuate nucleus (ARC) of sheep treated with vehicle (C), vehicle and stressful stimuli (S), allopregnanolone and stressful stimuli (AS) and allopregnanolone alone (A). Significance of differences: ab, *p* < 0.05 and AB, *p* < 0.01 vs. control group; cd, *p* < 0.05 and CD, *p* < 0.01 vs. stressed group.

**Figure 3:**
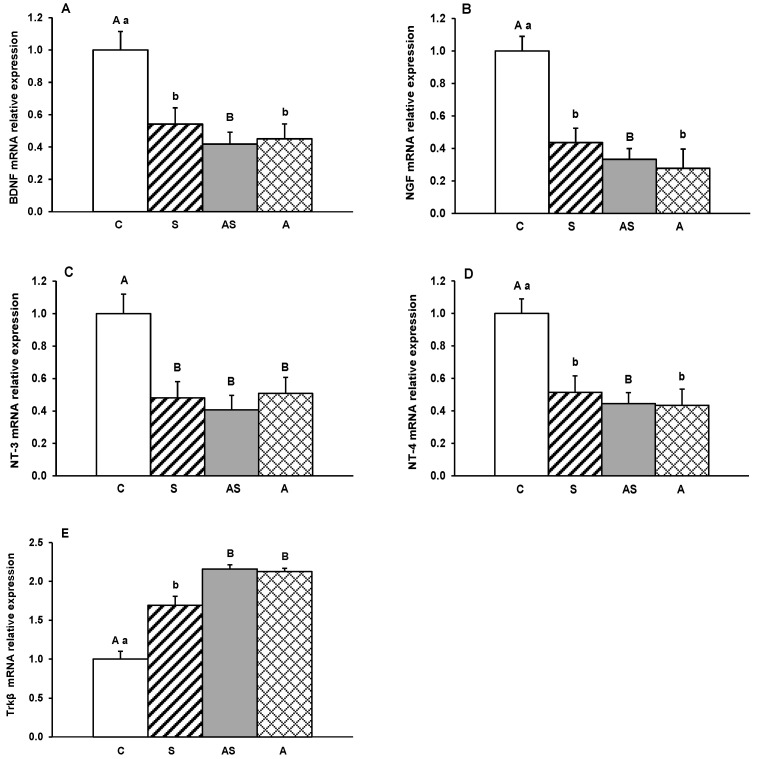
Relative mRNA abundance (mean ± SEM) of brain-derived neurotrophic factor (BDNF) (**A**), nerve growth factor (NGF) (**B**), neurotrophin-3 (NT-3) (**C**) and neurotrophin-4 (NT-4) (**D**) and tyrosine receptor kinase β (Trkβ) (**E**) in the anterior hypothalamus (AHA) of the of sheep treated with vehicle (C), vehicle and stressful stimuli (S), allopregnanolone and stressful stimuli (AS) and allopregnanolone alone (A). Significance of differences: ab, *p* < 0.05 and AB, *p* < 0.01 vs. control group.

**Figure 4:**
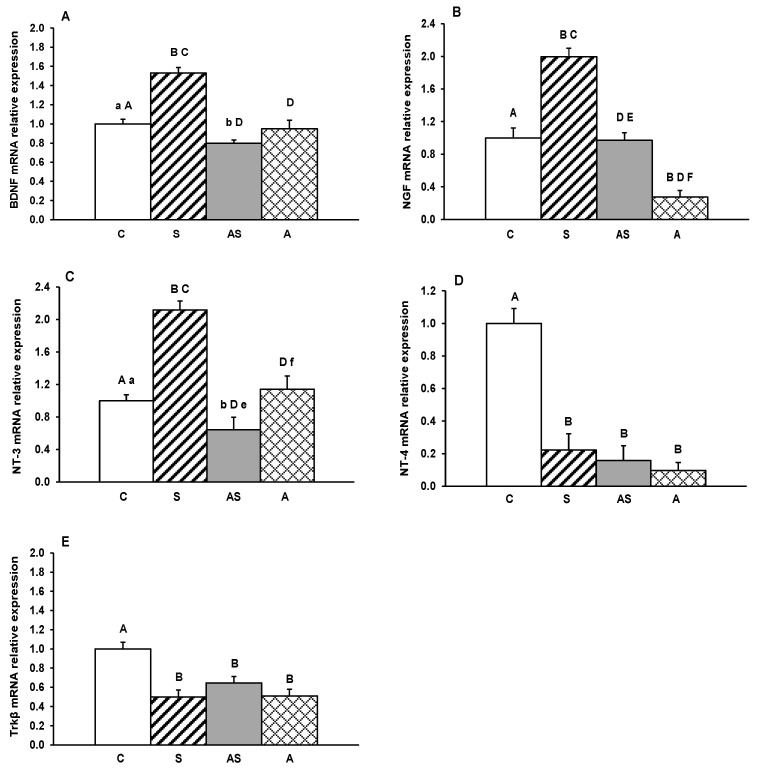
Relative mRNA abundance (mean ± SEM) of brain-derived neurotrophic factor (BDNF) (**A**), nerve growth factor (NGF) (**B**), neurotrophin-3 (NT-3) (**C**), neurotrophin-4 (NT-4) (**D**) and tyrosine receptor kinase β (Trkβ) (**E**) in the paraventricular nucleus (PVN) of the of sheep treated with vehicle (C), vehicle and stressful stimuli (S), allopregnanolone and stressful stimuli (AS) and allopregnanolone alone (A). Significance of differences: ab, *p* < 0.05 and AB, *p* < 0.01 vs. control group; CD, *p* < 0.01 vs. stressed group and ef, *p* < 0.05 and EF, *p* < 0.01 vs. allopregnanolone-stressed group.

**Figure 5:**
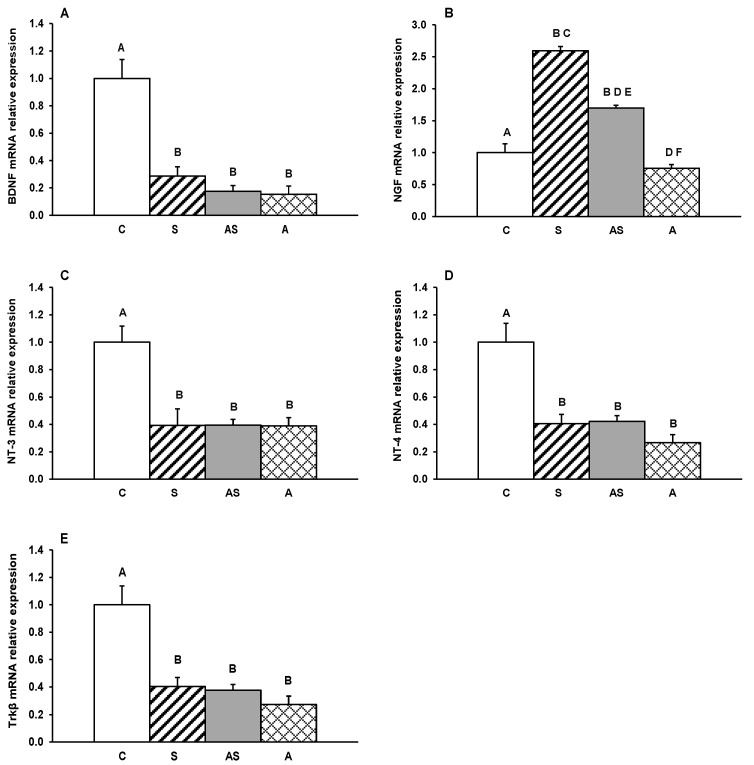
Relative mRNA abundance (mean ± SEM) of brain-derived neurotrophic factor (BDNF) (**A**), nerve growth factor (NGF) (**B**), neurotrophin-3 (NT-3) (**C**), neurotrophin-4 (NT-4) (**D**) and tyrosine receptor kinase β (Trkβ) (**E**) in the preoptic area (POA) of the of sheep treated with vehicle (C), vehicle and stressful stimuli (S), allopregnanolone and stressful stimuli (AS) and allopregnanolone alone (A). Significance of differences: AB, *p* < 0.01 vs. control group; CD, *p* < 0.01 vs. stressed group; EF, *p* < 0.01 vs. allopregnanolone-stressed group.

Gene transcripts for BDNF, its receptor Trkβ, and other neurotrophins (NGF, NT-3 and NT-4) were detected in all hypothalamic regions examined. Relative expression levels in the MBH, ARC, AHA, PVN and POA for all treatment groups are depicted in Figure 1, Figure 2, Figure 3, Figure 4 and Figure 5.

In the MBH, mRNA expression of all neurotrophins was significantly reduced by experimental treatments (*p* < 0.05–0.01, Figure 1A–D) in comparison to controls. BDNF and NGF showed a marked decrease in sheep receiving ALLO alone compared with both the stress-only group (*p* < 0.05) and, for NGF, the group exposed to stress combined with ALLO (*p* < 0.05). A decrease in Trkβ mRNA abundance was observed in stressed sheep, both with and without ALLO compared to controls (*p* < 0.05–0.01, Figure 1E). In contrast, Trkβ mRNA expression in the group receiving ALLO alone was higher (*p* < 0.01) than in animals subjected to stress alone and comparable to control levels.

In the ARC, BDNF mRNA levels increased significantly (*p* < 0.05–0.01, Figure 2A) in all experimental groups compared to the control group. For NGF and NT-3, only stressed sheep (Group S) had increased (*p* < 0.05) mRNA levels relative to controls. While ALLO administration did not affect NGF mRNA expression in groups AS and A, it significantly reduced NT-3 mRNA levels in these groups compared to controls (*p* < 0.05) and stressed sheep (*p* < 0.01). All experimental factors (Groups S, AS, and A) decreased the abundance of NT-4 mRNA compared to the control group (*p* < 0.05), with the most substantial reduction occurring in groups receiving ALLO (*p* < 0.05, AS and A vs. S, Figure 2D). Regarding Trkβ, stress alone suppressed (*p* < 0.01), and ALLO alone increased (*p* < 0.05) gene expression compared to controls (Figure 2E). In ALLO-infused sheep, both alone and in combination with stress, Trkβ mRNA levels were significantly higher (*p* < 0.01) than in stress-only group.

In the AHA, a significant decrease (*p* < 0.05–0.01) in relative mRNA levels of BDNF, NGF, NT-3 and NT-4 was observed in all experimental groups compared to the control group (Figure 3A–D). On the other hand, no significant differences were recorded between the experimental groups (S, AS, and A) for any individual neurotrophins. Conversely, Trkβ receptor mRNA expression was significantly increased (*p* < 0.05–0.01) in all treatment groups (S, AS, A) compared to controls (Figure 3E), with no differences recorded between the experimental groups.

In the PVN, the exposure of sheep for stress induced a significant (*p* < 0.01) increase in mRNA expression of BDNF, NGF and NT-3 compared to controls, as well as to the animals infused with ALLO in combination with stress (AS) or infused with ALLO alone (A) (Figure 4A–C). By contrast, allopregnanolone, administered alone (A) or with stress (AS), caused a decrease in BDNF (*p* < 0.05–0.01), NGF (*p* < 0.01) and NT-3 (*p* < 0.05–0.01) mRNA levels compared to controls and/or other groups. For NT-4, all experimental factors (S, AS and A) caused a significant (*p* < 0.01) decrease in mRNA abundance in the PVN compared to controls. Similar reductions in transcript levels were also observed for Trkβ (Figure 4E).

In the POA, mRNA expression levels of BDNF, NT3, NT4 and Trkβ decreased significantly (*p* < 0.01) in all experimental groups (S, AS and A) compared to the control group (Figure 5A,C–E). No significant differences were observed in transcript levels of individual neurotrophins in sheep exposed to stress (S), receiving ALLO with stress (AS), or ALLO alone (A). In contrast, NGF mRNA levels were significantly higher (*p* < 0.01) in sheep exposed to stress (S) and those administered ALLO with stress exposure (AS) compared to controls and ALLO-alone animals. Interestingly, ICV infusion of ALLO significantly counteracted (*p* < 0.01) stress-induced increase in NGF mRNA expression in the POA (Figure 5B).

## 3. Discussion

The hypothalamus integrates peripheral and environmental signals to maintain physiological and emotional homeostasis. It regulates the hypothalamic-pituitary neuroendocrine axis, autonomic nervous system, and key physiological processes such as feeding behavior and thermoregulation, fluid balance, circadian rhythms, emotional behavior, and reproductive function. By coordinating inputs from peripheral and central sources, the hypothalamus orchestrates adaptive responses essential for survival and well-being [35,37]. Additionally, adult neurogenesis in the hypothalamus, although less extensive than in the hippocampus or olfactory bulb, is increasingly recognized as an important mechanism in metabolic regulation, energy balance, and adaptive plasticity. Neural stem/progenitor cells, located mainly near the third ventricle, can give rise to new neurons in response to physiological cues such as diet, hormones, or stress [7]. Interestingly, new cell generation in the hypothalamus has also been confirmed in adult sheep, especially in the ARC and ME [38]. Neurotrophins play a pivotal role in this process by promoting the survival, differentiation and integration of newly formed neurons [39]. High levels of BDNF protein and/or BDNF mRNA have been reported in the hypothalamus of rodents and sheep [40,41]. The present study broadens current understanding of the presence and potential role of neurotrophins (BDNF, NGF, NT-3 and NT-4) in the sheep hypothalamus and investigates how severe emotional stress and ALLO, a stress response-related compound [42], affect their gene expression. It should be noted that the effectiveness of stress stimuli applied in the current study had previously been documented in the same animal model, demonstrating a significant activation of the HPA axis [42].

This type of stress exerted region-specific effects on neurotrophins expression in the sheep hypothalamus. BDNF transcript levels increased in the PVN and ARC, in contrast to other hypothalamic regions, such as the MBH, AHA and POA. The stress-induced rise in BDNF mRNA levels in these two hypothalamic nuclei may reflect their particular functions. The PVN is the central regulator of HPA axis activation, and the ARC is considered the primary site of hypothalamic neurogenesis. Previous studies in rats showed that acute immobilization stress caused a rapid increase in BDNF expression in the hippocampus and hypothalamus [43,44], preceding the activation of arginine-vasopressin (AVP)- and CRH-expressing neurons [45]. It has been suggested that BDNF may contribute to adaptive neuronal processes that support the allostatic load induced by stress [46]. Moreover, this neurotrophin shows high dynamic responsiveness to stress, with expression differing not only between brain regions but also depending on the type, duration, and frequency of the stressor. In rats, BDNF mRNA and protein levels showed rapid and significant increases in the PVN during acute (15–60 min to 2 h) and subchronic (2 h daily for 7 days) immobilization [43]. On the other hand, chronic immobilization stress (3 weeks, 3 h per day) reduced BDNF expression in the hypothalamus 60 min after the last stress session, but not after 180 min [46]. The effect of immobilization on BDNF immunostaining density and the number of BDNF-immunoreactive cells also exhibited duration-dependent effects. For instance, 30 min of stress exposure led to a decrease in BDNF-immunoreactive cells, whereas longer stress stimuli did not produce significant changes in the PVN [45]. The transient reduction in visible BDNF cells after brief stress could indicate protective neurotrophin release into the extracellular space to sustain neuronal activity. On the other hand, our results showed reduced BDNF expression after acute stress in the MBH, AHA, and POA, which does not exclude an initial increase in the neurotrophin levels. The impact of various stressors on these hypothalamic regions remains poorly understood and requires further studies. It is plausible that the regulation of distinct physiological functions at the hypothalamic level is subject to varying degrees of neurotrophic protection, potentially reflecting reduced energy allocation under unfavorable conditions. However, we previously demonstrated that acute stress significantly reduced both BDNF protein levels and gene transcript abundance in the CA1 and CA3 fields of the sheep hippocampus compared to the control group [47]. These findings indicate that the response of BDNF to this type of stress is structure-dependent and may follow two opposite trajectories: a transient increase during the initial phase as an adaptive or protective mechanism, or a decrease that could contribute to subsequent neuronal dysfunction. It has been shown that glucocorticoids (GCs) can suppress BDNF expression by binding to regulatory regions of the BDNF gene and recruiting repressive transcriptional complexes [48,49].

Stress exposure elicited also region-specific changes in Trkβ receptor mRNA: an increase was observed exclusively in the AHA area, while decreases occurred in other hypothalamic regions. Several reports have demonstrated the effect of stress on Trkβ in the PVN, hippocampus, and prefrontal cortex [47,50,51]. In rodents, acute stress fully activates the BDNF-Trkβ system, stimulating CRH neurons. High levels of phosphorylated Trkβ (p-Trkβ) were detected in CRH neurons, suggesting that autocrine BDNF-Trkβ signaling pathway may amplify HPA axis activation [25]. The decrease in Trkβ mRNA expression observed in the PVN in sheep is unexpected but may reflect increased p-Trkβ levels in the region. It should be noted that the sheep were exposed to stressors for 4 h before slaughter. Similar decreases in Trkβ transcript levels have also been reported previously in the hippocampus [47].

In addition to the BDNF-Trkβ system, our research demonstrated that the NGF mRNA expression pattern in response to acute stress was similar to that of BDNF, increasing in the PVN, ARC, and the POA. Immunofluorescence studies in adult and aged rats showed that neither acute (forced swim) nor chronic (high-light open field) stress altered the number of NGF-immunoreactive neurons in the PVN. However, the number of NGF-immunoreactive cells was significantly lower in older individuals (P720) compared to younger individuals after stress exposure, suggesting an aging-related attenuation of NGF signaling [27]. In psychosocial models of stress in male mice, including isolation and aggression, NGF mRNA and protein levels increased in the hypothalamus, especially in the PVN, but not in the hippocampus, cortex, or cerebellum, suggesting that local NGF synthesis supports BDNF in promoting neural plasticity in the adult brain and modulating HPA activity [52,53]. In a classic mouse model, isolation and male aggression also elevated NGF mRNA and protein in the POA region and ventrolateral nuclei of the hypothalamus [52,53].

Our results demonstrate that acute stress affects NT-3 and NT-4 mRNA expression in sheep hypothalamic areas. NT-3 transcript levels increased in the PVN and ARC but decreased in the MBH, AHA, and POA. Conversely, a decrease in NT-4 mRNA expression was observed in all hypothalamic regions studied. This is the first demonstration of NT-3 and NT-4 responses to acute stress in the sheep hypothalamus, as previous studies focused primarily on limbic structures such as the hippocampus and locus coeruleus (LC). Studies in rats have shown that chronic stress increases NT-3 transcription in the hippocampus, particularly in the dentate gyrus and neocortex [54,55], whereas acute stress does not, suggesting that NT-3, like BDNF and NGF, can be a specific marker of the hypothalamic adaptive response to certain stress situations. Moreover, the increase in NT-3 expression appears closely dependent on GC action, as adrenalectomy blocks this stress-induced effect [54]. Similar observations were made in the locus coeruleus, where chronic stress elevated NT-3 mRNA levels [54]. Interestingly, chronic stress also increased NT-3 concentration in peripheral tissues (salivary glands) and in blood plasma [56,57].

Numerous studies have indicated that ALLO influences neuroplasticity and function of neurotrophic factors, such as BDNF, and most of its effects are mediated through GABA_A_ and NMDA receptors [58]. This mechanism is particularly important in the context of stress response and emotional and neuroendocrine regulation. Activation of GABA_A_ receptors leads to hyperpolarization of neuronal membranes, causing reduced activity of CRH/AVP neurons in the hypothalamus, particularly in the PVN. Reduced activity of PVN neurons limits CRH secretion and consequently attenuates HPA response to stress [59]. Misztal et al. [42] demonstrated that central ALLO infusion decreased CRH mRNA expression in the PVN and POMC mRNA expression in the AP of stressed sheep. In addition, a decrease in CRH concentration in ME perfusates, as well as ACTH and cortisol levels in blood, were observed. These findings [42] suggest that ALLO may act centrally as a suppressor of HPA axis activity in stressed sheep. Moreover, ALLO is considered a potential therapeutic agent for mental disorders associated with HPA axis dysfunction and reduced BDNF levels, such as depression and anxiety disorders [60]. The present study on sheep showed that a temporary increase in ALLO decreased the abundance of all neurotrophins gene transcripts in most hypothalamic regions. Moreover, in the PVN, this neurosteroid specifically counteracted stress-induced upregulation of BDNF, NGF, and NT-3 mRNA expression. Similar effects were also observed in stressed sheep for NGF and NT-3 transcripts in the ARC, as well as for NGF mRNA in the POA. By inhibiting rapid neurotrophic response, ALLO may play an important protective role, preventing excessive neuronal activity during the critical phase of acute stress. Therefore, ALLO released concurrently with HPA axis activation during acute stress [59,61] appears to modulate impulsive neuronal excitation within specific hypothalamic nuclei. Comparable changes in neurotrophins expression in response to ALLO administration were also previously described in hippocampal CA1 and CA3 regions [47]. In rodent studies, social isolation resulted in decreased hippocampal ALLO and BDNF levels, whereas ALLO administration prevented anxiety-depressive behaviors, impaired neurogenesis, and reduced BDNF expression [62].

In the present study, the increase in BDNF transcript levels in the ARC, as well as elevated Trkβ mRNA abundance in both the ARC and AHA in response to ALLO alone is particularly noteworthy. The former may be related to hypothalamic neurogenesis, a process occurring in sheep in the ARC and ME [36]. Interestingly, newborn hypothalamic neurons in sheep have a longer maturation period than in rats, and the distribution pattern of neuroblasts in the sheep hypothalamus is comparable to that of humans. Infantes-Lopes et al. [32] were the first to demonstrate that acute stress could reduce the proliferation of immature neuronal cells in the parenchymal areas surrounding the 3rd ventricle of the hypothalamus. These alterations were accompanied by an increase in stress-responsive microglia and exacerbated inflammation, as evidenced by elevated IL-6 levels. Decreased hypothalamic neurogenesis is considered to be strongly associated with altered hypothalamic functions, therefore preservation of ALLO-induced neurotrophic activity in the ARC seems particularly relevant. Conversely, the observation that ALLO stimulates Trkβ expression may indicate increased neuronal sensitivity to reduced BDNF levels in critical situations. Such upregulation of Trkβ expression may be an important regulatory mechanism allowing antidepressants to remain effective even when BDNF levels are low [63,64,65]. Casarotto et al. [10] demonstrated that certain antidepressants directly bind to the Trkβ receptor, facilitating its synaptic localization and subsequent activation by BDNF. Similarly, Shirayama et al. [66] reported that ALLO alone may exert antidepressant effects via BDNF-Trkβ signaling. In a rat learned helplessness depression model, intracerebroventricular ALLO administration produced antidepressant effects that were abolished by co-infusion of the Trkβ inhibitor ANA-12.

## 4. Materials and Methods

### 4.1. Animal Management

The experiment involved 24 2-year-old Polish Longwool female sheep selected to represent reproductive seasonality. Animals were housed at the Sheep Breeding Center of the Kielanowski Institute of Animal Physiology and Nutrition, Polish Academy of Sciences in Jablonna near Warsaw, Poland (52° N, 21° E), under natural lighting conditions. Sheep were fed twice a day with a pelleted concentrate formulated according to their physiological status and the recommendations of the National Research Institute of Animal Production (Krakow-Balice, Poland) and National Institute for Agricultural Research (France) [67]. Hay, water, and mineral licks were available ad libitum. During the experimental period, sheep were kept in individual pens that permitted visual, olfactory, and tactile contact with other animals.

### 4.2. Brain Surgery and Experimental Design

Sheep were implanted with a stainless steel guide cannula (outer diameter: 1.2 mm) into the third ventricle (IIIv) using stereotaxic coordinates (frontal 30.5–31.5 mm; sagittal 1.0 mm) based on the ovine hypothalamic atlas [68]. The procedure was performed under general anesthesia induced by intravenous administration of xylazine (40 mg/kg), xylapan, and ketamine (10–20 mg/kg; Bioketan, Vetoquinol Biowet, Puławy, Poland). A cranial bore hole was drilled to allow cannula insertion according to the established method of Traczyk and Przekop [69]. The guide cannula was fixed to the skull with stainless steel screws and dental cement, and the external opening of the canal was sealed with a stainless steel cap. Postoperatively, ewes received daily antibiotic treatment for 5 days (streptomycin 1 g and benzylpenicillin 1,200,000 IU; Polfa, Warszawa, Poland) and analgesics for 4 days (metamizole sodium 50 mg/animal; Biovetalgin, Biowet Drwalew, Drwalew, Poland, or meloxicam 1.5 mg/animal; Metacam, Boehringer Ingelheim, Ingelheim am Rhein, Germany). Correct cannula placement in the IIIv was verified by cerebrospinal fluid (CSF) outflow during surgery and after slaughter. All animals included in the study had confirmed correct cannula localization.

The experiment was performed during the reproductive season, from mid-October to mid-December, after estrus synchronization using the Chronogest-CR method [70]. Sheep were randomly divided into 4 groups (*n* = 6 each) and treated over 3 consecutive days of the late luteal phase (days 12–14) of the estrous cycle as follows: (i) intracerebroventricular (ICV) infusion of vehicle for 3 days (C group); (ii) ICV infusion of vehicle and exposure to stressful stimuli on day 3 (S group); (iii) ICV infusion of ALLO and exposure to stressful stimuli on day 3 (AS group); and (iv) ICV infusion of ALLO alone for 3 days (A group). ALLO (Sigma-Aldrich, St Louis, MO, USA) was dissolved in a 1:1 mixture of dimethyl sulfoxide (DMSO; Blirt, DNA-Gdańsk, Gdańsk, Poland) and 20% 2-hydroxypropyl-β-cyclodextrin (Sigma-Aldrich, St Louis, MO, USA), and diluted in a Ringer-Locke (RL) solution after 24 h. The control vehicle consisted of the same mixture without ALLO, diluted in RL (2% DMSO). All infusions were administered in four 30-min sessions at 30-min intervals (from 10:00 to 14:00 h), using a BAS Bee microinjection pump (Bioanalytical Systems Inc., West Lafayette, IN, USA) and calibrated 1.0-mL gas-tight syringes. This intermittent infusion protocol, previously validated in our ovine model [42], maintains bioactive concentrations while preventing continuous receptor saturation. The selected dose of ALLO (4 × 15 μg/60 μL/30 min/day) was based on our preliminary results (Grant No. 2015/19/B/NZ9/03706) and previous studies [42,47]. Stress exposure included psychological stress (isolation from other sheep) and physical stress (partial movement restriction in the experimental cage) and lasted for the 4-h infusion period. During treatments, sheep were kept in comfortable experimental cages, with prior acclimatization ensuring normal behavior.

### 4.3. Brain Tissue Collection

Directly after the experiment, sheep were slaughtered following pharmacological stunning (xylazine 0.2 mg/kg body weight and ketamine 3 mg/kg body weight, intravenously). The brains were promptly removed from the skull. After separation of the ME, each brain was sectioned sagittally into cerebral hemispheres. Hypothalamic blocks (cut to a depth of 2 mm) were dissected into the following regions: the MBH, with separated ARC; AHA, with separated PVN; and POA [71], according to the stereotaxic atlas of the ovine brain [68]. Landmarks included the optic chiasm, thalamus and mammillary body. All dissections were performed on sterile glass plates placed on ice, and the collected structures were immediately frozen in liquid nitrogen and stored at −80 °C.

### 4.4. Total RNA Isolation, cDNA Synthesis and Quantitative Real-Time Polymerase Chain Reaction Analysis

Total RNA from the hypothalamic and AP tissues was isolated using the NucleoSpin RNA II kit (MACHEREY-NAGEL GmbH and Co., Düren, Germany) in accordance with the manufacturer’s protocol. RNA quality and quantity were determined spectrophotometrically (NanoDrop ND-1000, Thermo Fisher Scientific, Waltham, MA, USA). The RNA integrity was evaluated electrophoretically by separation on a 1.5% agarose gel containing ethidium bromide. For complementary DNA (cDNA) synthesis, 1500 ng mL^−1^ of mRNA from the each selected area of the hypothalamus in a total volume of 20 μL, was retro transcribed using a TranScriba Kit (A&A Biotechnology, Gdynia, Poland) in a 20 µL reaction volume according to the manufacturer’s protocol. Quantitative real-time PCR was performed using 5 × HOT FIREPol^®^ EvaGreen qPCR Mix Plus (Solis BioDyne, Tartu, Estonia). Each 15-µL reaction contained 2 µL of cDNA template, 1 µL of primers (0.5 µL each, 10 pmol/mL), 3 µL of buffer PCR Master Mix and 9 µL of dd H_2_O. The cycling conditions were as follows: initial denaturation at 95 °C for 15 min, denaturation at 95 °C for 15 s, annealing at 60 °C for 20 s, and elongation at 72 °C for 20 s (40 cycles), then 1 cycle at 72 °C for 7 min (product stabilization). The melting curve was performed over 70–95 °C at 0.5 °C intervals. Negative controls without the cDNA template were included in each reaction. The identity of each PCR products was confirmed by direct sequencing (Genomed, Warsaw, Poland). Specific primers for the target genes: *BDNF*, *Trkβ*, *NGF*, *NT-3* and *NT-4*, as well as the reference genes: glyceraldehyde-3-phosphate dehydrogenase (*GAPDH*) and peptidylprolyl isomerase C (*PPIC*) were designed using Primer3 software v.4.1.00 (The Whitehead Institute, Boston, MA, USA) (Table 1) and synthesized by Genomed (Warsaw, Poland). Amplification specificity was further validated by electrophoresis of the PCR products on a 2% agarose gel and visualized under a UV light camera. Data were analyzed using Rotor Gene 6000 software 1.7 software (Qiagen, Hilden, Germany) with the comparative quantification option and evaluated using the Relative Expression Software Tool (2008) (REST 2009 v.1) according to Pfaffl et al. (2002) [72] based on a PCR efficiency correction algorithm developed by Pfaffl et al. (2004) [73]. Gene expression levels were normalized to the geometric mean of *GAPDH* and *PPIC* expression to compensate for cDNA concentration variation and PCR efficiency between individual samples.

### 4.5. Statistical Analyses

Data normality was initially assessed using the Shapiro–Wilk test to distinguish parametric from non-parametric variables. Differences in mRNA expression levels of BDNF, NGF, NT-3, NT-4 and Trkβ in all hypothalamic regions and treatment groups were analyzed using non-parametric methods, including the Kruskal–Wallis test with multiple comparisons of average ranks, followed by pairwise comparisons with the Mann–Whitney U test for individual groups (STATISTICA v. 13.3, Stat Soft, Tulsa, OK, USA). Differences were considered significant at *p* < 0.05, and all data are presented as mean ± standard error of the mean (SEM).

## 5. Conclusions and Limitations

The present results demonstrate that acute stress differentially modifies neurotrophins mRNA expression in distinct hypothalamic regions, including increased neurotrophic activity in specific nuclei. The predominant inhibitory effect of ALLO on neurotrophin synthesis, particularly under conditions of acute stress, may help prevent excessive neuronal activation. On the other hand, ALLO-induced upregulation of the BDNF-Trkβ system in the ARC may indicate a positive relationship between this neurosteroid and hypothalamic adult neurogenesis. In addition to the significant conclusions that can be drawn from this study, it is imperative to consider several inherent limitations when interpreting the results. The analysis concentrated exclusively on the mRNA expression levels of selected neurotrophins, without concurrent assessment of their corresponding protein levels. Despite the objective of the present study being to evaluate changes at the transcriptomic level, given the possibility of discrepancies between transcript expression and translation, a certain degree of caution should be exercised. Furthermore, the present study was conducted exclusively in female sheep at a specific stage of the reproductive cycle. Due to the significant influence of estrous phase and sex on hormonal balance and the neuroendocrine response to stress, it is evident that the results of this study cannot be extrapolated directly to males or other stages of the reproductive cycle. Subsequent research is required to investigate the complex mechanisms that differentiate between the two sexes, which should cover a broader spectrum of brain structures and analyses at the cellular and functional levels. A further significant limitation is the fact that the process of hypothalamic neurogenesis in sheep is still poorly understood, and our study shows only a possibility of neurotrophin response to stress and associated neurosteroid activity. In summary, these limitations do not diminish the significance of the presented results but rather point to directions for future research. Given the growing interest in neurological research on sheep [36,74], translating the findings of studies conducted on sheep to humans may be more rational than in the case of rodents.

## Figures and Tables

**Table 1:** Specific primers sequences.

Gene	Primers (5′–3′)	Genbank Acc. No.	Amplicon Size
*BDNF*	F: CGTTGGCTGACACTTTTGAA R: CGCAGCATCCAGGTAATTTT	XM_012143442.1	188
*NGF*	F: CAGTCCAAGGGGCTGGAT R: AGTGTGGCCAGGACAGAAAG	XM_004002369.5	101
*NT-3*	F: TGCCACGATCTTACAGGTGA R: TGCCTGGATCAGCTTGATTA	XM_004006944.5	151
*NT-4*	F: CCTGAGATGTCACGAAGGAC R: TGAACACCTGTCAGCACCTC	XM_027978595.3	112
*TRKβ*	F: TGTCTGAGCTGATCCTGGTG R: TATCTGCAGGTTTGCCAGTG	XM_012117231.2	155
*GAPDH*	F: GGGTCATCATCTCTGCACCT R: GGTCATAAGTCCCTCCACGA	NM_001190390.1	131
*PPIC*	F: TGGAAAAGTCGTGCCCAAGA R: TGCTTATACCACCAGTGCCA	XM_004008676.1	158

## Data Availability

The datasets analyzed during the current study are available from the corresponding author upon reasonable request.
